# Association between Breast Cancer Knowledge and Mammogram Utilization among Immigrant Muslim Arab Women in California: Cross-Sectional Design

**DOI:** 10.3390/healthcare10122526

**Published:** 2022-12-14

**Authors:** Sarah Alkhaifi, Hanan Badr

**Affiliations:** Maternity and Child Health Department, Faculty of Nursing, King Abdulaziz University, Jeddah 21589, Saudi Arabia

**Keywords:** breast cancer, mammogram, Arab American, immigrant women

## Abstract

Background: Regular mammogram screenings have contributed to early breast cancer (BC) diagnoses and lowered the mortality rate by 40% in the United States of America (USA). Nonetheless, ethnic women living in developed countries, such as immigrant Muslim Arab women (IMAW), are less likely to get mammograms. Aim of the study: In our study, we aimed to understand health behaviors among IMAWs as understudied populations in the USA. Methods: We conducted a cross-sectional study on a convenience sample of IMAW living in southern California. We used logistic regression and multivariate logistic regressions to analyze the data. Results: The total number of participants who completed the survey was 184 IMAW. Participants who had a higher level of knowledge about BC signs and symptoms and mammogram knowledge were more likely to have obtained a mammogram at some point compared with their counterparts (OR = 1.23, *p* = 0.03, CI: 1.07–1.42; OR = 2.23, *p* = 0.23, CI: 1.11–4.46, respectively). Conclusions: Our results provide more evidence emphasizing the important influence of BC and mammogram knowledge on immigrant women’s behavior regarding mammogram utilization. The average level of knowledge in all three domains (BC risk factors, BC signs and symptoms, and mammogram use) reported in this study is considered low.

## 1. Introduction

Breast cancer (BC) is the most common cancer, and it is the second leading cause of death among women, killing about 685,000 women per year globally [1]. Regular mammogram screenings have contributed to early BC diagnoses and lowered its mortality rate by 40% from 1987 to 2017 in the United States of America (USA) [2]. Nonetheless, ethnic women living in developed countries, such as immigrant Muslim Arab women (IMAW), are less likely to undergo mammograms [3,4].

Few researchers have attempted to evaluate mammogram screening rates among IMAW. Those who have reported biennial mammogram rates lower than the Healthy People 2030 target, which is 77.1% [5]. Ayyash et al. [6] and Schwartz et al. [7] reported that in the preceding two years, 58% (*n* = 365) and 51% (*n* = 102) of IMAW, respectively, had undergone a mammogram. A significant proportion of the immigrant Muslim women in Chicago were IMAW, with a biannual rate of 37% (*n* = 240) [8]. According to Hasnain et al. [9], 35% of immigrant Arab women (*n* = 207) underwent mammograms within the previous two years.

Knowledge about BC and its screenings was found to have an impact on Arab and Muslim women’s behaviors regarding mammograms. However, these populations were, in general, found to have inadequate knowledge of BC. In qualitative studies, the women reported that BC could be caused by breast trauma, infection, the evil eye, God’s will, not drinking enough camel milk, or not getting enough sun exposure [10,11,12,13]. Additionally, women reported that a mammogram was a diagnostic procedure and should be recommended when signs and symptoms appeared. Some IMAW even believed that radiation from mammograms could increase their risk of developing BC [11,12,13]. Misunderstandings or inadequate knowledge about BC and mammograms could have resulted in delayed screening among these populations.

Studies that quantitively explored the relationship between immigrant Arab and Muslim women’s level of knowledge about BC and BC screenings and their mammogram use are scarce. To date, only three studies (in California, Illinois, and Michigan) have revealed positive associations between immigrant Muslim and Arab women’s levels of knowledge and whether they have ever obtained a mammogram, the regularity with which they obtain mammograms, or their intention to obtain a mammogram [6,14,15]. Our results expand the body of the literature by exploring the association between the level of knowledge of BC signs and symptoms, BC risk factors and mammograms, and IMAW’s mammogram utilization in California.

Moreover, we are filling the gap in understanding health behaviors among Arab and Muslim immigrants as understudied populations in the USA. Despite Arab and Muslim people having lived in the USA since 1875, published health-related research on this population is scarce [16]. We conducted our study in California, which is home to the largest population of Arabs (both Muslim and Christian) in the USA [17,18]. To the best of our knowledge, no study has examined the association between BC and mammogram knowledge and IMAW’s mammogram utilization in California.

### Theoretical Framework

In our research, we were guided by a socioecological model that has been used to guide several studies and national programs targeting health promotion and disease prevention. According to the socioecological model, there is a range of factors that influence individuals’ well-being and health behaviors, including intrapersonal, interpersonal, community, and organizational factors. Intrapersonal factors, such as knowledge, influence individuals’ behaviors toward disease prevention. Based on the theoretical underpinning of intrapersonal factors, IMAW’s level of knowledge about BC and mammograms has an impact on their mammogram utilization. Hence, our hypothesis is as follows: Compared to their peers, IMAW who claim higher levels of BC and mammography awareness will be more likely to have received mammograms within the past two years.

## 2. Materials and Methods

### 2.1. Study Design

This is a cross-sectional study conducted on a convenience sample of IMAW.

### 2.2. Recruitment and Data Collection

Our study was approved by the University of California, Los Angeles institutional review board (IRB #19-001971). Due to the COVID-19 pandemic, recruitment and data collection were conducted via social media using two private groups on Facebook and three WhatsApp groups for immigrant Arab and Muslim women living in Southern California. The primary investigator contacted the administrator of each group to post an announcement of the study information, including the study’s introduction and aim along with links to the survey in Arabic and English. Data were collected between March 2020 and August 2020.

Qualtrics was used for data collection. We obtained study consents and conducted screening electronically. Participants were screened by answering questions to determine their eligibility for the study. Enrollment was limited to women who (a) were 45 years or older, (b) self-identified as Muslim, (c) lived in California, (d) had immigrated from an Arab country, (e) had not been previously diagnosed with BC, and (f) were able to speak Arabic or English. The reasoning behind including woman who were 45 years or older was based on the age recommended by the American Cancer Society in 2020 for starting regular mammograms. Women who did not meet the inclusion criteria were automatically prevented from participating in the study.

### 2.3. Instruments

Both Arabic and English surveys included (a) demographic questions, such as the age of the participants, education level, working status, and ability to read and write in English; (b) history of mammogram utilization; (c) a modified breast cancer awareness measure; and (d) level of knowledge about mammograms.

#### 2.3.1. Mammogram History

The two main outcomes in the study were measured as self-reported: (a) having ever had a mammogram and (b) having had a mammogram in the previous two years.

#### 2.3.2. Breast Cancer Level of Knowledge

A modified breast cancer awareness measure (breast CAM) was used to measure the level of knowledge about breast cancer. The tool includes two subscales: (a) a BC signs and symptoms subscale (10 items) and (b) a BC risk factors subscale (10 items). Each item has three response options: “Yes”, “No”, and “I do not know”. The scoring system is (1) for each correct answer and (0) for “each incorrect answer or if the participant reported they do not know the information”. For each subscale, the score ranges from 0 to 10 points. A higher score indicates a higher level of knowledge about BC signs and symptoms and BC risk factors [19]. 

The tool was modified and translated into Arabic and was used among Arab women in Oman by Al-Khasawneh et al. in 2016 [19]. The tool demonstrated an acceptable level of relatability. The breast cancer signs and symptoms subscale and breast cancer risk factors subscale reported excellent reliability (α = 0.89 and 0.85, respectively). The tool also reported robust criterion validity (R = 0.58, *p* < 0.01) among Omani women [19]. 

#### 2.3.3. Mammogram Level of Knowledge

The primary investigator developed three questions to measure IMAW’s level of knowledge about mammograms. These questions were as follows: Is mammography a useful tool for the early detection of breast cancer? Can mammography be performed on any X-ray machine? Which of the following techniques is the most effective way to detect breast cancer at an early stage?

The questions were viewed by a researcher who holds a PhD degree and has research experience focused on mammogram utilization among immigrant populations in the USA. Additionally, after translating the three questions into Arabic, fourteen immigrant Arab women reviewed the questions for clarity. According to the reviewers, no changes were required. 

To calculate the score for mammogram level of knowledge, each question has three response options. Questions one and two may be answered by “Yes”, “No”, or “I do not know”. Zero points are assigned to the wrong answer or “I do not know” for the breast CAM scoring. For question three, a point is assigned to the correct answer “mammogram”, and zero points are assigned to “breast self-examination” or “clinical breast examination”. Thus, the maximum score in this section is three points; the minimum score is zero. Higher scores indicate a higher level of knowledge about mammogram screening. These three questions were translated into Arabic by a professional translator and then evaluated by two bilingual nurses (Arabic and English) who hold PhD degrees in nursing. 

### 2.4. Data Analysis

SPSS 26.0 was used for data analysis. Descriptive analyses (percentage, mean, range, standard deviation) were used to summarize sample demographic characteristics. The associations between the outcomes (mammogram history) and level of BC and mammogram knowledge were measured by logistic regression. Cronbach’s alpha was also calculated for each subscale in the breast CAM.

Data analysis involved several steps. In the first step, simple bivariate logistic regressions were run to examine the association between each outcome: (a) having ever had a mammogram and (b) having had a mammogram during the previous two years; and all of the independent variables: (a) BC signs and symptoms, (b) BC risk factors, and (c) mammogram knowledge.

In the second step, multivariate logistic regressions were conducted to evaluate the association between each outcome and each independent variable, controlling for selected sociodemographic covariates. If a covariate had a *p* ≤ 0.2 and was linked to each outcome, it was included in the regression model. Three covariates were included for the first outcome (if the participants ever had a mammogram). These covariates were age, income, and length of residence in the United States. For the second outcome (having had a mammogram within the previous two years), two covariates were included in the multivariate analysis: income and length of residence in the United States. To avoid multicollinearity, age at immigration was not included as a covariate for both outcomes because it was highly correlated with length of residency in the USA (*r* = −0.8, *p* < 0.01).

## 3. Results

### 3.1. Sample Characteristic

The total number of participants who completed the survey was 184 IMAW. The average age of the participants was (M = 50.4, SD = 5.58); 60.9% were between the ages of 45 and 50, and 39.1% were aged 51 years or older. All the participants were married; 139 (76.2%) of the participants had two or more children, 30 (16.4%) had one child, and 12 (6.6%) did not have any children.

Regarding employment and annual income, 90 (49%) of the participants were not working, 56 (30.7%) had a part-time job, and 30 (16.4%) had a full-time job. Even though the number of full-time working women was not high, 74 (40.2%) of the women reported an income of more than USD 55,000, and 176 (95.7%) reported that they had health insurance.

The vast majority of participants had immigrated from Syria, Jordan, or Palestine, and about 34% of participants had been in the United States for 11 to 20 years. The participants’ ages at the time of immigration ranged between 5 and 45 years, and the mean was (M = 31, SD = 9.2).

Most of the participants, 159 (86.4%), had obtained a mammogram at least once in their lifetimes, and 124 (67.4%) reported that they had not had a mammogram within the previous two years. Table 1 presents the other demographic characteristics of the study’s sample.

The women revealed average levels of knowledge about BC risk factors (M = 4.1, SD = 2.8) and BC signs and symptoms (M = 6.4, SD = 1.6). In our results, each subscale demonstrates acceptable levels of internal consistency reliability. The Cronbach’s alpha for BC signs and symptoms and BC risk factors was 0.80 and 0.61, respectively (Table 2).

### 3.2. Association between BC and Mammogram Levels of Knowledge and Ever Having Obtained a Mammogram

Data analysis showed a positive correlation between mammography use and knowledge level. Bivariate analysis revealed that participants who knew more about the symptoms and signs of BC were more likely than their peers to have ever had a mammogram (OR = 1.23, *p* = 003, CI: 1.07–1.42; Table 3). Additionally, participants who had more awareness of mammograms were twice as likely to have ever undergone a mammogram than their counterparts (OR = 2.23, *p* = 023, CI: 1.11–4.46; Table 3). In the multivariate models, while controlling for annual income, length of residence in the USA, and age, both predictors remained significant for ever having had a mammography (Table 4, Models 2 and 3).

### 3.3. Association between BC and Mammogram Levels of Knowledge and Having Had a Mammogram within the Last 2 Years

High levels of mammography knowledge were found to be positively associated with participants having had a mammogram during the past two years in the bivariate analysis (OR = 1.99, *p* = 0.023, CI: 1.24–3.22; Table 3). The likelihood that a participant had a mammogram within the previous two years increased by 12% for participants with knowledge of BC signs and symptoms with each additional unit of knowledge (OR = 1.12, *p* = 0.01, CI: 1.01–1.25). Finally, participants were more likely to have had screening within the previous two years if they had higher levels of knowledge about BC risk factors (OR = 1.92, *p* = 0.013, CI: 1.13–3.25; Table 3). All three predictors remained significant for the second outcome after annual income and time of residency in the United States were taken into account in the multivariate analysis (Table 5, Models 1–3).

## 4. Discussion

Our study results provide more evidence emphasizing the importance of BC and mammogram knowledge to immigrant women’s behavior regarding mammogram utilization. The average level of knowledge in all three domains (BC risk factors, BC signs and symptoms, and mammogram use) we explored in this study is considered low. Other studies conducted on immigrant Arab and Muslim women reported similar levels of knowledge [6,20,21]. Williams et al. [21] reported a low level of knowledge among immigrant Arab women for BC screenings. Study participants also included African American women (*n* = 116) and Latina women (*n* = 112); however, the immigrant Arab women (*n* = 112) reported the lowest scores regarding mammography [21]. Notably, the researchers did not measure any association between level of knowledge and mammogram utilization.

Findings from our study suggest a positive association between levels of knowledge and mammogram utilizations among IMAW, aligning with findings reported by Badr et al. [14], who found that Lebanese American women (Muslim and Christian) who reported a higher knowledge of mammogram screening guidelines had increased odds of having had a mammogram at least once [14]. In the Arab world, Muslim and Arab women in Jordan and Palestine who reported higher levels of BC knowledge were more likely to have obtained a mammogram within the last two years compared with their counterparts [22,23]. In Yemen, low levels of BC knowledge were negatively associated with Muslim and Arab women’s mammogram use [24].

High levels of knowledge about BC risk factors predicted that a participant would have had a mammogram within the last two years. It appeared that participants who were aware of factors that increased their risk of BC were more likely to obtain mammograms regularly. Researchers could speculate that a high level of knowledge about BC risk factors may have also increased participants’ perceptions of BC susceptibility, a health belief that a person is at risk for a certain disease, which may have increased their mammogram use. Therefore, further studies could explore the relationship between the level of BC knowledge and health beliefs related to mammogram screenings.

All three multivariate models displayed a significant negative association between annual income and mammogram history (Table 4, Models 1–3). When controlling for knowledge levels (each domain separately) and selected demographic variables, participants who reported an annual income of <USD 16,000 were less likely to have ever had a mammogram compared with those who reported an annual income of >USD 55,000. These findings may suggest that although the level of knowledge is an important variable affecting mammogram utilization, other demographic factors may also impact IMAWs’ levels of knowledge and, consequently, their mammogram utilization. Our findings are too limited to expand on that conjecture. Therefore, future studies must examine the effect of knowledge levels on IMAWs’ mammogram use, taking their sociodemographic contexts into account.

As shown in Table 5, Model 3, levels of knowledge about mammograms and the number of years spent in the USA predicted whether participants had obtained a mammogram within the last two years. It is worth mentioning that other studies also reported a positive association between duration of residency in the USA and mammogram utilization. A longer residency in the USA was not only associated with the likelihood that immigrant Arab and Muslim women would have had at least one mammogram but it was also associated with the likelihood that women would adhere to a mammogram screenings schedule [8,9,13,25].

Additionally, scholars could speculate, based on a finding from a qualitative study by Islam et al. [26], that participants who had spent a longer time in the USA may have been subjected to BC educational campaigns to enhance their level of knowledge, resulting in enhanced adherence to mammograms. This suggests a gap for future researchers to explore in more detail—the possibility that level of knowledge is a mediator between duration of residency in the USA and mammogram utilization among IMAW.

## 5. Limitations

We must consider several limitations when interpreting our findings. Selection bias may have resulted from recruiting participants via social media, and women may have been exposed to virtual BC educational campaigns, which may have enhanced their level of knowledge. Thus, the actual level of knowledge among IMAW about BC and mammograms may be lower than what is reported in our results. Additionally, about 60% of the participants reported having a bachelor’s degree. Hence, results from this study may not be generalizable for women with low levels of education. Finally, the three items developed by the researcher to measure participants’ levels of knowledge about mammograms have not been psychometrically tested. Thus, findings regarding mammogram knowledge levels should be interpreted with caution.

## 6. Implications

According to the results from this study, IMAW demonstrated low levels of BC and mammogram knowledge. These findings suggest the need for interventions to improve the level of knowledge among IMAW. Higher knowledge levels in all three domains (BC risk factors, BC signs and symptoms, and mammogram use) predicted that the women had received mammograms within the last two years, suggesting that these interventions may help to enhance women’s adherence to mammogram screenings. Interventions should be tailored to women’s social and cultural contexts to assure their acceptability and effectiveness.

## Figures and Tables

**Table 1:** Sample characteristics (*n* = 184).

Characteristics	Frequency (*n*)	Percentage %
Country of origin *		
Egypt	22	12
Jordan	44	24
Palestine	34	18.5
Syria	46	25
Other countries	38	20.5
Length of stays in USA *		
≤10 years	49	26.7
11 to 20 years	62	33.8
21 to 30 years	53	28.8
>30 years	20	10.7
Age of women at the time of immigration *		
≤31 years	109	59.2
>31 years	75	40.8
Education **		
High school and below	22	12.1
Diploma or associate degree	53	29.1
Bachelor’s degree and higher	107	58.8
Annual income ***		
<USD 16,000	28	16.2
USD 16,001–35,000	38	22.0
USD 35,001–55,000	32	18.6
>USD 55,000	74	43.0
Length of marriage (in years) *		
≤10	21	11.4
11–20	58	31.5
21–30	74	40.3
≥31	31	16.8
Speaking proficiency in English (self-rated) **		
Excellent	65	35.7
Good	100	55
Poor	17	9.3
Reading proficiency in English (Self rated) **		
Excellent	76	41.8
Good	89	48.9
Poor	17	9.3

*n = 184, ** *n* = 182, *** *n* = 172.

**Table 2:** Descriptive analysis and Cronbach’s alpha of breast cancer awareness measure (breast CAM) (*n* = 184).

Scale/Subscales	Range	Mean	SD	Cronbach’s Alpha
Breast Cancer signs and symptoms Breast Cancer risk factors Mammogram knowledge	0–10 0–10 0–3	6.4 4.1 1.6	1.6 2.8 0.71	0.80 0.61

**Table 3:** Association between BC and mammogram level of knowledge (BC and mammogram) and mammogram utilizations (*n* = 184).

Variables	Ever Obtained a Mammogram OR (CI)	Obtained Mammogram within the Last Two Years OR (CI)
Breast cancer risk factors, *n* = 184	1.19 (0.91–1.55)	1.29 * (1.05–1.58)
Breast cancer Signs and symptoms, *n* = 184	1.23 ** (1.07–1.42)	1.12 * (1.01–1.25)
Knowledge about mammogram, *n* = 184	2.23 * (1.11–4.46)	1.99 * (1.24–3.22)

* *p* < 0.05, ** *p* < 0.01.

**Table 4:** Association between level of knowledge (BC and mammogram) and ever having had mammogram in IMAW, controlling for covariates ^1^ (Models 1–3, *n* = 172) ^1^.

Variables	Model 1 OR (CI)	Model 2 OR (CI)	Model 3 OR (CI)
Breast cancer risk factors level of knowledge Breast cancer signs and symptoms level of knowledge Mammogram level of knowledge	1.16 (0.87–1.53)	1.23 ** (1.05–1.44)	2.48 * (1.12–5.46)
Annual income <USD 16,000	0.212 * (0.05–0.85)	0.23 * (0.05–0.98)	0.30 * (0.06–1.33)
USD 16,000–35,000	0.59 (0.15–2.23)	0.45 (0.11–1.82)	0.755 (0.19–3.00)
>USD 35,000–55,000	0.274 * (0.07–0.982)	0.25 * (0.06–0.92)	0.34 (0.09–1.28)
>USD 55,000	Reference	Reference	Reference
Age	1.12 (1.00–1.26)	1.10 (0.97–1.24)	1.13 * (1.01–1.28)
Length of residence in the USA	1.015 (0.95–1.07)	1.02 (0.96–1.08)	1.01 (0.96–1.07)

* *p* < 0.05, ** *p* < 0.01, ^1^ Each model includes one main independent variable, controlling for covariate.

**Table 5:** Association between level of knowledge (BC and Mammogram) and having had a mammogram in the previous two years in IMAW controlling for covariates ^1^ (Models 1–3, *n* = 172).

Variables	Model 1 OR (CI)	Model 2 OR (CI)	Model 3 OR (CI)
Breast cancer risk factors level of knowledge Breast cancer signs and symptoms level of knowledge Mammogram level of knowledge	1.26 * (1.02–1.57)	1.12 * (1.01–1.26)	1.92 * (1.13–3.25)
Annual income <USD 16,000 USD 16,000–35,000 >USD 35,000–55,000 >USD 55,000	0.46 (0.16–1.27) 0.787 (0.31–1.96) 0.64 (0.25–1.63) Reference	0.44 (0.16–1.24) 0.66 (0.26–1.63) 0.57 (0.22–1.45) Reference	0.58 (0.19–1.68) 0.88 (0.35–2.21) 0.71 (0.27–1.86) Reference
Length of residence in the USA	1.03 (0.99–1.07)	1.03 (0.97–1.01)	1.05 * (0.98–1.12)

* *p* < 0.05, ^1^ Each model includes one main independent variable, controlling for covariates.

## Data Availability

The data are available from the authors upon request.
